# Effects of Interleukin-1β Inhibition on Blood Pressure, Incident Hypertension, and Residual Inflammatory Risk: A Secondary Analysis of CANTOS

**DOI:** 10.1161/HYPERTENSIONAHA.119.13642

**Published:** 2019-12-30

**Authors:** Alexander MK Rothman, Jean MacFadyen, Tom Thuren, Alastair Webb, David G Harrison, Tomasz J. Guzik, Peter Libby, Robert J. Glynn, Paul M. Ridker

**Affiliations:** 1 From the Department of Cardiology, Chesterman Cardiothoracic Unit, Northern General Hospital, Sheffield, United Kingdom (A.M.K.R.); 2 Department of Infection, Immunity and Cardiovascular Disease, University of Sheffield, United Kingdom (A.M.K.R.); 3 Center for Cardiovascular Disease Prevention (J.M., R.J.G., P.M.R.), Brigham and Women’s Hospital, Harvard Medical School, Boston, MA; 4 Cardiovascular Division (P.L.), Brigham and Women’s Hospital, Harvard Medical School, Boston, MA; 5 Novartis Pharmaceutical Corporation, One Health Plaza, East Hanover, NJ (T.T.); 6 Centre for Prevention of Stroke and Dementia, Department of Clinical Neurosciences, University of Oxford, United Kingdom (A.W.); 7 Vanderbilt University, Nashville, TN (D.G.H.); 8 Institute of Cardiovascular and Medical Research, Queen Elizabeth University Hospital, University of Glasgow (T.J.G.); 9 Department of Medicine, Jagiellonian University, School of Medicine, Cracow, Poland (T.J.G.).

**Keywords:** blood pressure, diagnosis, inflammation, interleukins, myocardial infarction

## Abstract

While hypertension and inflammation are physiologically inter-related, the effect of therapies that specifically target inflammation on blood pressure is uncertain. The recent CANTOS (Canakinumab Anti-inflammatory Thrombosis Outcomes Study) afforded the opportunity to test whether IL (interleukin)-1β inhibition would reduce blood pressure, prevent incident hypertension, and modify relationships between hypertension and cardiovascular events. CANTOS randomized 10 061 patients with prior myocardial infarction and hsCRP (high sensitivity C-reactive protein) ≥2 mg/L to canakinumab 50 mg, 150 mg, 300 mg, or placebo. A total of 9549 trial participants had blood pressure recordings during follow-up; of these, 80% had a preexisting diagnosis of hypertension. In patients without baseline hypertension, rates of incident hypertension were 23.4, 26.6, and 28.1 per 100-person years for the lowest to highest baseline tertiles of hsCRP (*P*>0.2). In all participants random allocation to canakinumab did not reduce blood pressure (*P*>0.2) or incident hypertension during the follow-up period (hazard ratio, 0.96 [0.85–1.08], *P*>0.2). IL-1β inhibition with canakinumab reduces major adverse cardiovascular event rates. These analyses suggest that the mechanisms underlying this benefit are not related to changes in blood pressure or incident hypertension.

**Clinical Trial Registration—:**

URL: https://clinicaltrials.gov. Unique identifier: NCT01327846.

## Abstract 2

Supplemental Digital Content is available in the text.

**See Editorial, pp 297–298**

Hypertension and inflammation are physiologically inter-related.^1^ In observational epidemiological studies, raised inflammatory biomarkers such as hsCRP (high sensitivity C-reactive protein) and IL (interleukin)-6 correlate with increased blood pressure^2–4^ and left ventricular dysfunction,^5^ and predict the future development of hypertension,^6^ heart failure,^5^ and major adverse cardiovascular events.^2^ Yet, the pathophysiologic mechanisms through which inflammation and elevated blood pressure interact, and their causal relationships, remain uncertain. Preclinical evidence suggests that elevated blood pressure is associated with a proinflammatory state mediated, in part, by cytokines, such as IL-1β, that alter endothelial, immune, and central nervous system responses potentiating the development of hypertension.^1^ For example, IL-1β is increased in the kidneys of mice with angiotensin II–induced hypertension,^7^ and activation of IL-1 receptor 1 enhances renal sodium transporter activity resulting in salt retention.^8^ In mouse models genetic deletion of IL-1 receptor 1,^9^ pharmacological blockade of IL-1 signaling,^10^ and administration of an IL-1β neutralizing antibody therapy^11^ have been demonstrated to reduce blood pressure. Downstream of IL-1, IL-6, and CRP are implicated in the development of hypertension through angiotensin II^12–14^ and central nervous system-mediated T-cell activation^15^ and vascular inflammation.^1^ Immune cell infiltration and their release of inflammatory cytokines like IL-1β have not only been associated with blood pressure elevation but also with end-organ damage associated with hypertension.^16^ Despite this evidence, the effect of therapies that specifically target inflammation on blood pressure is largely unknown.

In the recent CANTOS (Canakinumab Anti-inflammatory Thrombosis Outcome Study), canakinumab—a fully human monoclonal antibody targeting IL-1β—significantly reduced rates of recurrent cardiovascular events^17^ and hospitalization for heart failure^18^ in patients with a history of myocardial infarction and a persistent proinflammatory response. Furthermore, while lipid levels did not change in CANTOS, the magnitude of cardiovascular benefit associated with canakinumab was related directly to the magnitude of inflammation inhibition achieved as detected by on-treatment reductions in hsCRP and IL-6.^19,20^ Per protocol, all CANTOS participants had blood pressure systematically measured before randomization and throughout trial follow-up. CANTOS thus afforded the unique opportunity to test formally whether IL-1β inhibition reduces blood pressure, prevents the development of incident hypertension, or modifies relationships between hypertension and cardiovascular events.

## Methods

The data from the study is not available to other researchers.

### Study Design and Participants

CANTOS was a randomized, double-blind placebo-controlled trial that evaluated 3 doses of canakinumab (50, 150, or 300 mg) administered subcutaneously once every 3 months as compared with matching subcutaneous placebo for the prevention of major adverse atherosclerotic events.^17,21^ Between April 28, 2011, and March 3, 2014, CANTOS enrolled 10 061 patients with a history of myocardial infarction and concentrations of hsCRP of 2 mg/L or greater from over 1000 clinical sites in 39 countries. The study excluded patients with a history of chronic or recurrent infections, previous malignancy other than basal cell skin carcinoma, a suspected or known immunocompromised state, or a history of (or high risk for) tuberculosis or HIV-related disease, and those using systemic anti-inflammatory treatments. All participants provided written informed consent to participate in the trial, which was monitored by an independent data and safety monitoring board.

### Procedures

Clinical history including cardiovascular risk factors and a preexisting diagnosis of hypertension was documented by enrolling physician before randomization. A diagnosis of incident hypertension was made in patients with no prior history of hypertension and a blood pressure of >140/90 during follow-up.

Investigators were instructed to record resting, seated blood pressure in triplicate after the subject had been sitting for at least 5 minutes with back supported and both feet placed on the floor before drug administration at baseline and 3, 6, and 12 months using an appropriately sized blood pressure cuff with a validated automated device or a manual sphygmomanometer. Blood pressure for each visit represents the mean of triplicate readings rounded to the nearest integer for systolic and diastolic measurements.

Blood samples were obtained from all trial participants in placebo and canakinumab groups at randomization and at 3 months before repeat canakinumab (or placebo) injection.^19^ Samples were assayed for hsCRP and samples from selected sites assayed for IL-6 concentrations in a central laboratory as previously described.^19,20^

### Outcomes

The primary end point of CANTOS was a composite of adjudicated recurrent myocardial infarction, stroke, or cardiovascular mortality. The key prespecified secondary cardiovascular end points included these events as well as adjudicated episodes of hospitalization for unstable angina requiring urgent coronary revascularization. Additional major end points adjudicated by the trial end point committee included cardiovascular mortality, cancer mortality,^22^ heart failure hospitalization,^18^ and all-cause mortality. Median follow-up was 3.7 years.

### Statistical Analysis

For the current analyses, study participants were divided into 4 quartiles according to systolic blood pressure at study enrolment (baseline). χ^2^ tests were used to assess for significant differences between these quartiles for categorical variables, and Wilcoxon rank-sum tests for continuous variables. Cox proportional-hazard models stratified according to time since the index myocardial infarction and trial part were used to evaluate whether increasing levels of hsCRP associated with incident hypertension during trial follow-up in patients with no preexisting diagnosis of hypertension and to estimate relative hazards for major adverse cardiovascular events, myocardial infarction, coronary revascularization, stroke, heart failure hospitalization, and all-cause mortality in treatment groups as compared with those allocated placebo in all study participants. Analyses compared the time to clinical event and adjusted for characteristics known to influence hsCRP, including age, sex, body mass index, LDL (low-density lipoprotein)-cholesterol, and diabetes mellitus.

The trial is registered.

### Role of the Funding Source

The trial was sponsored by Novartis Pharmaceuticals. The sponsor was responsible for data collection. The corresponding author had full access to all study data and was responsible for the decision to submit for publication.

## Results

A total of 9549 of the 10 061 patients randomized in CANTOS had blood pressure data available at baseline and at 3 months. At enrolment, a preexisting diagnosis of hypertension was documented in 79.6% of patients with a population median blood pressure of 130/79. Stratified by quartile of baseline systolic blood pressure (quartiles 1–4: <120, 120–129, 130–140, and >140 mm Hg) the upper quartile was older with higher lipid levels (total, LDL and HDL [high-density lipoprotein] cholesterol, and triglycerides) and higher rates of diabetes mellitus, hypertension, stroke, and antihypertensive therapy (Table 1). In contrast, smoking was more common in the lowest quartile of baseline systolic blood pressure, as was a prior diagnosis of heart failure and ST-segment–elevation myocardial infarction (compared to non–ST-segment–elevation myocardial infarction, Table 1).

**Table 1.**
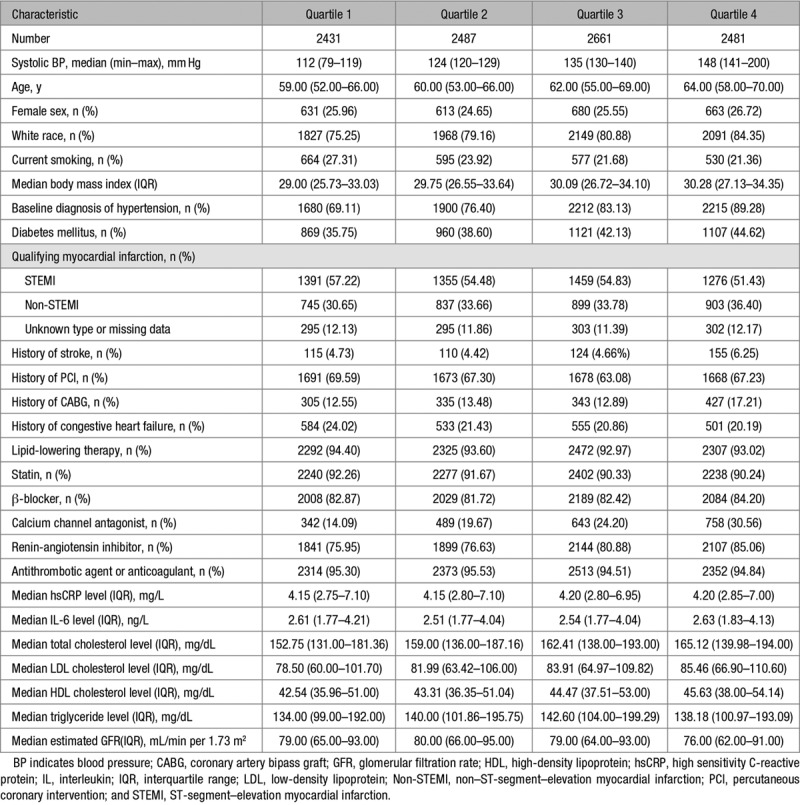
Patient Demographics Stratified by Quartile of Baseline Systolic BP

**Figure 1.**
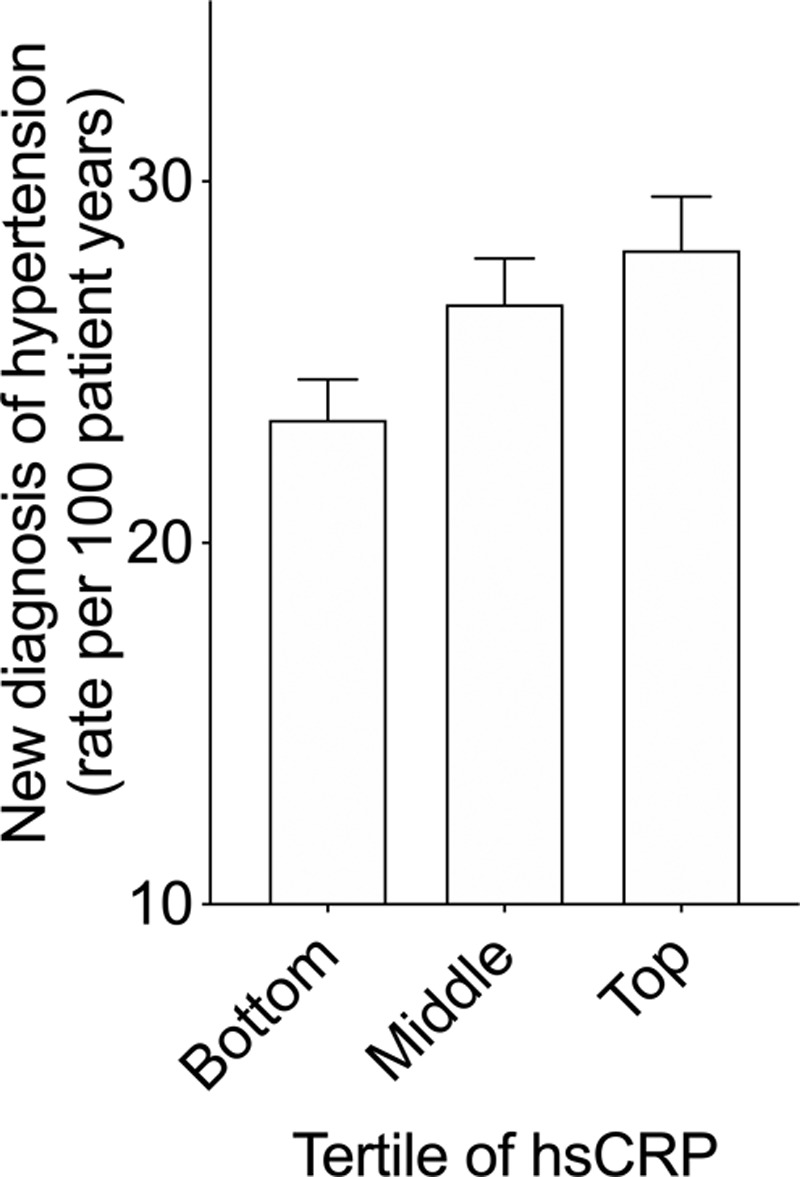
Incident hypertension by tertile of baseline hsCRP (high sensitivity C-reactive protein; rate per 100-person years ± 95% CI, Cox proportional-hazard compared the time to diagnosis of incidence of hypertension and adjusted for age, sex, and body mass index, *P*>0.2).

Baseline concentrations of the inflammatory markers hsCRP and IL-6 were similar across quartiles of systolic blood pressure at baseline (Table 1). In patients without a preexisting diagnosis of hypertension, increased baseline hsCRP associated with modestly higher rates of incident hypertension during follow-up, but this effect was not statistically significant (hsCRP tertile 1, 23.4 per 100 person-years [21.1–25.9]; tertile 2, 26.6 [24.1–29.4]; tertile 3, 28.1 [25.4–31.0], *P*>0.2, Figure 1).

**Figure 2.**
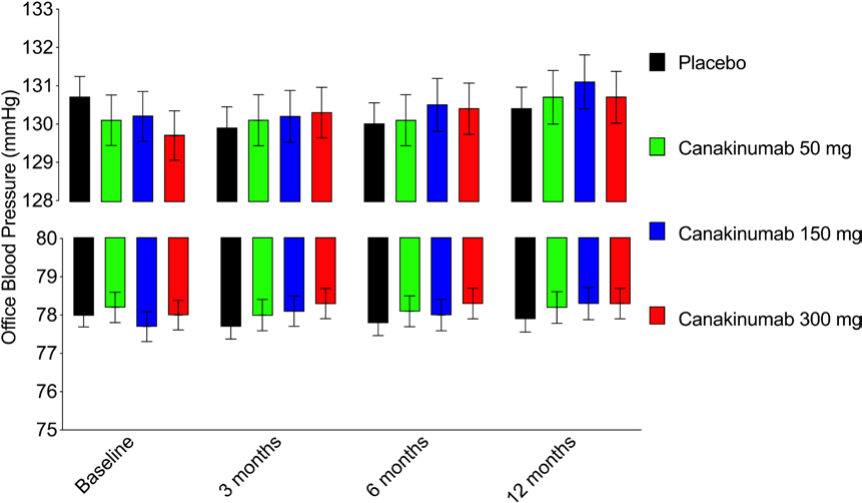
Lack of effect of canakinumab compared to placebo on blood pressure. Office blood pressure of patients randomly allocated to canakinumab (50 mg, 150 mg, and canakinumab 300 mg) or placebo at baseline, 3, 6, and 12 months (mean and 95% CI, upper: systolic blood pressure, lower: diastolic blood pressure.

In the complete study group, random allocation to canakinumab as compared to placebo did not alter recorded blood pressure between baseline and the 3-, 6-, or 12-month visits overall or individually at the 50, 150, or 300 mg doses of active therapy (all *P*>0.2, Figure 2, and Figure S1 in the online-only Data Supplement). In analyses limited to those with normal blood pressure at trial entry, canakinumab as compared to placebo did not reduce the rate of new hypertension diagnoses (hazard ratio, 0.96 [0.85–1.08], *P*>0.2). Furthermore, no reduction in blood pressure was identified in patients randomly allocated to canakinumab who achieved a greater than median reduction in inflammation as assessed by on-treatment concentrations of hsCRP and IL-6 (Table 2).

**Table 2.**
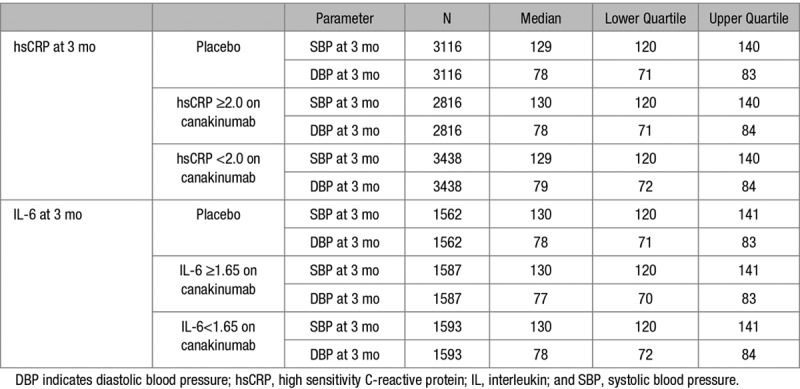
SBP and DBP at 3 Months in the Placebo Group and in the Combined Canakinumab Treatment Group Stratified by Achieved Levels of hsCRP and IL-6 Above or Below the On-Treatment Study Median

**Figure 3.**
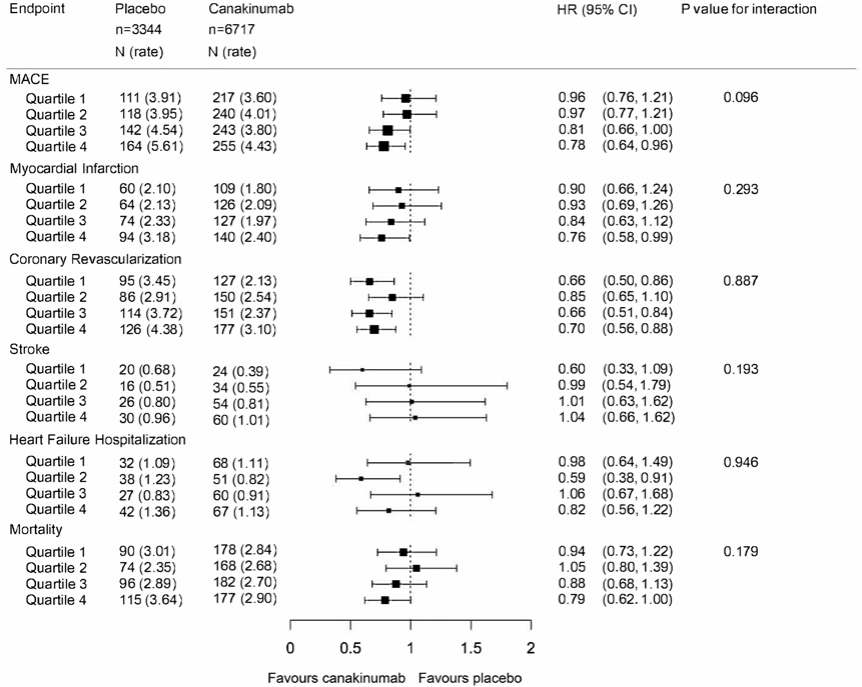
The effect of canakinumab on clinical events stratified by baseline systolic blood pressure. Major adverse cardiovascular events (MACE), myocardial infarction, coronary revascularization, stroke, heart failure hospitalization, and mortality adjusted for age, sex, body mass index, LDL (low-density lipoprotein)-Cholesterol, and diabetes mellitus (placebo n=3344, canakinumab n=6717, number of events indicated by box size, hazard ratio indicated by box position with 95% CI, Q1: lowest baseline systolic blood pressure, Q4: highest baseline systolic blood pressure, <1 favors canakinumab). HR indicates hazard ratio.

As previously published, random allocation to canakinumab resulted in a significant reduction in the composite primary end point of nonfatal myocardial infarction, nonfatal stroke, or cardiovascular mortality.^17^ As would be anticipated, when stratified by baseline systolic blood pressure, major adverse cardiovascular events, recurrent myocardial infarction, coronary revascularization, stroke, and mortality were more frequent among trial participants with increasing levels of baseline systolic blood pressure (Figure 3). Although formal tests for interaction were not significant, in patients with a systolic blood pressure of ≥130 mm Hg and baseline, allocation to canakinumab was associated with an absolute risk reduction of 3.4% compared to 1.8% in patients with a baseline systolic blood pressure <130 mm Hg (Figure 3). These differences may, however, be due to play of chance. No significant benefit of canakinumab on stroke was observed at any quartile of blood pressure.

## Discussion

These analyses of the randomized, double-blind, placebo-controlled CANTOS trial are inconsistent with prior evidence in that in patients with normal blood pressure, those with raised hsCRP did not have increased rates of incident hypertension. Per protocol patients with a hsCRP of <2 mg/L were excluded from trial entry and as such the truncated range of hsCRP levels may contribute to this finding. As anticipated, higher levels of baseline blood pressure in CANTOS associated with higher vascular risk during trial follow-up in both placebo and canakinumab groups. However, random allocation to canakinumab, a drug that inhibits IL-1β and reduces both IL-6 and hsCRP, did not reduce the development of incident hypertension nor reduce blood pressure at 3, 6, or 12 months.

In atherosclerotic mice, genetic deletion of IL-1 receptor 1^9^ can reduce blood pressure and administration of an IL-1β neutralizing antibody produces a dose-dependent reduction in lesion formation.^11^ In humans, a reduction in blood pressure accompanied a 14-day treatment with anakinra (an IL-1 receptor antagonist which inhibits both IL-1α and β) following acute coronary syndrome in post hoc analysis of the 182 in the Medical Research Council Interleukin-1 Receptor Antagonist Heart study.^23–25^ Yet, as reported here in 9549 CANTOS subjects, none of the 3 doses of canakinumab evaluated compared with placebo altered blood pressure at 3, 6, or 12 months. Potential explanations of these apparent differences include sample size, duration of follow-up, different approaches to IL-1 inhibition and distinct inclusion criteria. CANTOS enrolled patients with stable atherosclerosis and a prior myocardial infarction whereas MRC ILA Heart enrolled patients following an acute coronary syndrome. Perhaps most significantly a prior diagnosis of hypertension and prior myocardial infarction was present in only 30% and 24% of patients in MRC ILA Heart compared to 80% and 100% of patients in CANTOS, respectively. As such, the proportion of patients prescribed antihypertensive medication at baseline was higher in CANTOS.

In rats with angiotensin II–induced vascular injury and hypertension, it has been shown that mycophenolate mofetil prevents renal damage^16^ and interferon-γ targeting reduces cardiac arrhythmias and fibrosis without significant effects on blood pressure.^26^ As such, in patients with hypertension, anti-inflammatory therapy may provide benefit without blood pressure reduction. Consistent with previous literature and with clinical practice, absolute rates of adverse events in CANTOS increased with baseline systolic blood pressure in patients randomly allocated to both canakinumab or placebo. As previously reported, the 150 mg dose of canakinumab reduced the risk of the composite primary end point in CANTOS by 15% compared to placebo (3.86 versus 4.50 events per 100 person-years).^17^ Although not statistically significant, random allocation to canakinumab reduced the composite primary end point by 16% in patients with a baseline systolic blood pressure of 130 to 140 mm Hg (3.80 versus 4.54 events per 100 person-years) and 21% in patients with a baseline systolic blood pressure >140 mm Hg (4.43 versus 5.61 events per 100 person-years).

### Strengths and Limitations

As a post hoc analysis of a randomized controlled trial, these data may be prone to type I error. Yet, despite our very large sample size, our analysis detected no interaction between canakinumab efficacy and blood pressure level nor any effect on measured blood pressure itself. This analysis was also limited by the total number of cardiovascular events after stratification by baseline systolic blood pressure, especially for less frequent outcomes such as stroke. By contrast, strengths of our study include its large sample size and consistency of results across follow-up timepoints and each dose of canakinumab.

### Conclusions

While inhibition of IL-1β with canakinumab reduces cardiovascular event rates, these analyses suggest that the mechanisms underlying this benefit are not related to changes in blood pressure or incident hypertension.

### Perspectives

Hypertension and inflammation are physiologically inter-related. Yet, the mechanisms through which inflammation and elevated blood pressure interact, and their causal relationships, remain uncertain. CANTOS afforded the unique opportunity to test whether IL-1β inhibition reduced blood pressure, prevented the development of incident hypertension, or modified relationships between hypertension and cardiovascular events. While inhibition of IL-1β with canakinumab reduced cardiovascular event rates, these analyses suggest that the mechanisms underlying this benefit are not related to changes in blood pressure or incident hypertension.

## Sources of Funding

The investigator-driven CANTOS (Canakinumab Anti-inflammatory Thrombosis Outcomes Study) trial was funded by Novartis. A.M.K. Rothman and A. Webb are supported by Clinical Research Career Development Fellowships from the Wellcome Trust (A.M.K. Rothman: 206632/Z/17/Z; A. Webb: 206589/Z/17/Z).

## Disclosures

None.

## Supplementary Material

## Novelty and Significance

### What Is New?

- In patients with prior myocardial infarction and persistent proinflammatory response: Canakinumab reduced cardiovascular events but not blood pressure or the rate of incident hypertension.

### What Is Relevant?

- Inhibition of IL (interleukin)-1β does not alter blood pressure or incident hypertension.

### Summary

While inhibition of IL-1β with canakinumab reduces cardiovascular event rates, the mechanisms are not related to changes in blood pressure or incident hypertension.
