# Supplementary figures and images for: Effects of Interleukin-1β Inhibition on Blood Pressure, Incident Hypertension, and Residual Inflammatory Risk: A Secondary Analysis of CANTOS
Source: Hypertension. 2019 Dec 30;75(2):477–82. doi: 10.1161/HYPERTENSIONAHA.119.13642 (PMC7055941; doi:10.1161/HYPERTENSIONAHA.119.13642)

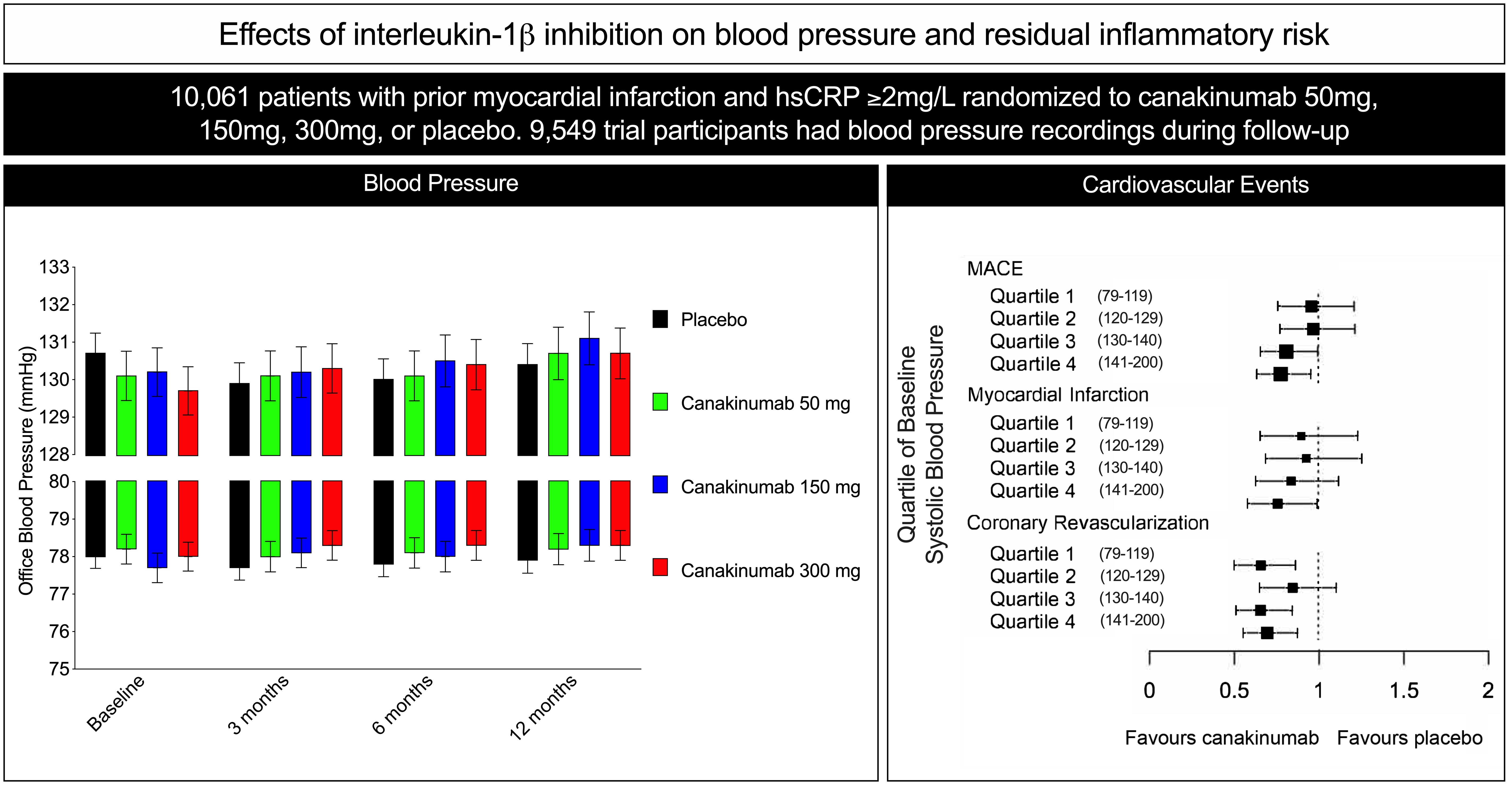

Supplement: Supplementary file 1 [file hyp-75-477-s001.jpg]
