# Supplementary material for: Effects of Interleukin-1β Inhibition on Blood Pressure, Incident Hypertension, and Residual Inflammatory Risk: A Secondary Analysis of CANTOS
Source: Hypertension. 2019 Dec 30;75(2):477–82. doi: 10.1161/HYPERTENSIONAHA.119.13642 (PMC7055941; doi:10.1161/HYPERTENSIONAHA.119.13642)
Supplement: Supplementary file 2 [file hyp-75-477-s002.docx]

Effects of interleukin-1β inhibition on blood pressure, incident hypertension, and residual inflammatory risk: a secondary analysis of CANTOS.

Alexander M K Rothman,^1^ Jean MacFadyen,^2^ Tom Thuren,^3^ Alastair Webb,^4^ David G Harrison,^5^ Tomasz J. Guzik,^6^ Peter Libby,^7^ Robert J Glynn,^2^ Paul M Ridker^2^

Short title: Effects of interleukin-1β inhibition on blood pressure

1. Department of Cardiology, Chesterman Cardiothoracic Unit, Northern General Hospital, Sheffield, UK, S5 7AU and Department of Infection, Immunity & Cardiovascular Disease, University of Sheffield, Sheffield, UK, S10 2RX
2. Center for Cardiovascular Disease Prevention, Brigham and Women’s Hospital, Harvard Medical School, 900 Commonwealth Avenue, Boston, MA 02215, USA
3. Novartis Pharmaceutical Corporation, One Health Plaza, East Hanover, NJ 07936
4. Centre for Prevention of Stroke and Dementia, Department of Clinical Neurosciences, University of Oxford, UK, OX3 9DU
5. Director of Clinical Pharmacology, Room 536 Robinson Research Building, Vanderbilt University, Nashville, TN 37232-6602
6. Institute of Cardiovascular and Medical Research, Queen Elizabeth University Hospital, University of Glasgow, 120 University Place, Glasgow G12 8TA and Department of Medicine, Jagiellonian University, School of Medicine, Cracow, Poland
7. Cardiovascular Division, Brigham and Women’s Hospital, Harvard Medical School, 75 Francis Street, Boston, MA

Keywords: Inflammation, hypertension, interleukin-1b, hsCRP, canakinumab, intervention

Corresponding author: Paul M. Ridker

Tel: +1 617 732 8790 Fax: +1 617 734 1508 Email: pridker@bwh.harvard.edu

Text:

Supplemental Tables:

Supplemental Figures:


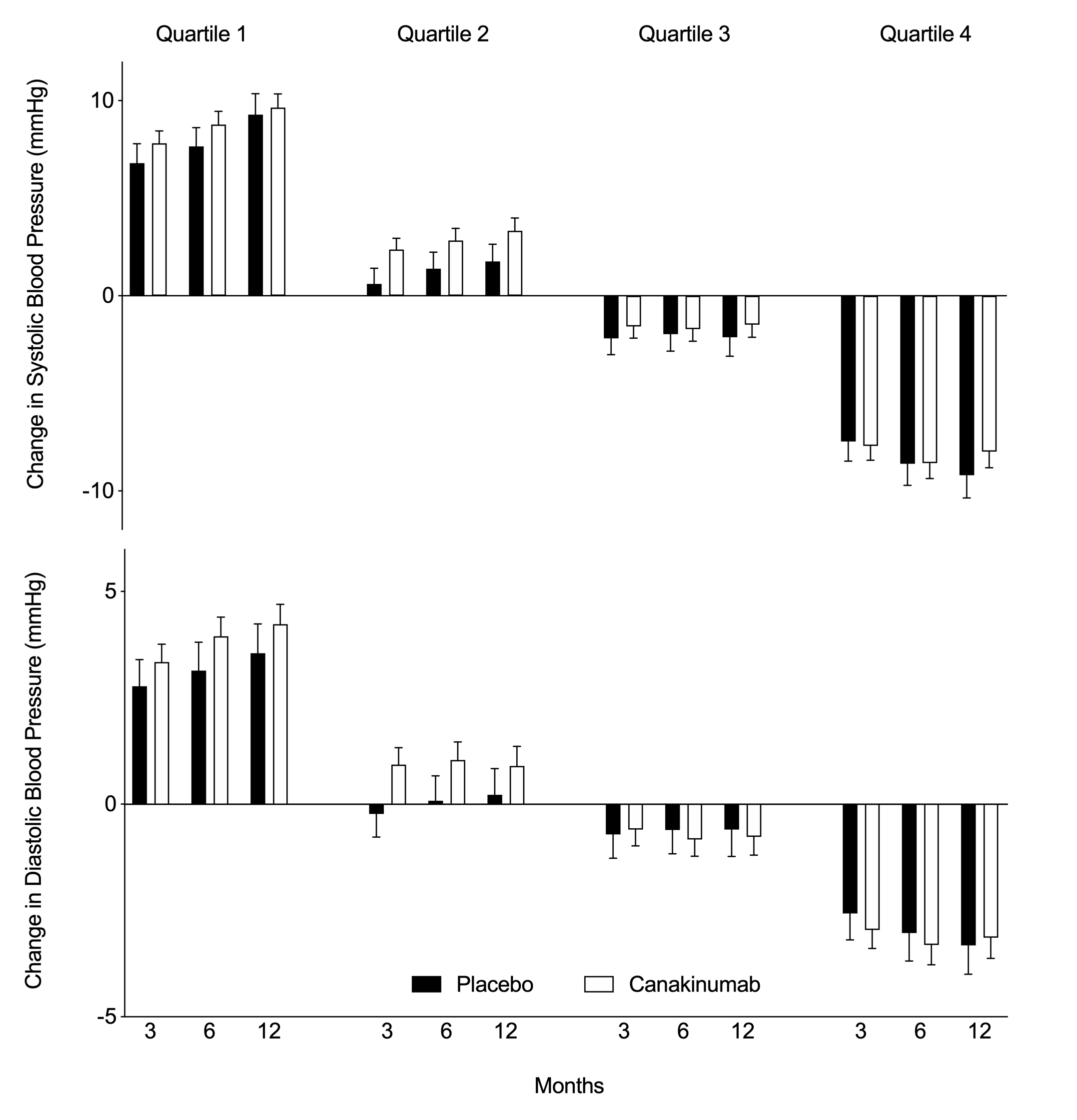


Figure S1: Change in systolic and diastolic blood pressure from baseline stratified by quartiles of baseline systolic blood pressure. Placebo is compared to all active groups of canakinumab at 3-, 6- and 12-months (mean and 95% confidence interval).
